# Association between LDL‐C/ApoB Ratio and Cognitive Impairment: a retrospective study of NHANES

**DOI:** 10.1002/alz70856_096766

**Published:** 2025-12-24

**Authors:** Wenzhi Shi, Peiyao Wei, Juanjuan Lu, Shan Huang, Rui Li, Xingzhi Guo

**Affiliations:** ^1^ Shaanxi Provincial People's Hospital, Xi’an, Shaanxi Provincial, China

## Abstract

**Background:**

Elevated levels of ApoB and LDL‐C have been associated with CI^1^. The LDL‐C/ApoB ratio is a marker of LDL particle size, with smaller, denser LDL (sdLDL) particles being more atherogenic and a risk factor for cardiovascular diseases^2^. However, the relationship between LDL‐C/ApoB and CI remains unclear.

**Method:**

This study utilized data from the NHANES covering the years 2011 to 2014, including 1,105 participants over 60 years old. To investigate the relationship between LDL‐C/ApoB and CI, the logistic regression analysis was employed. Furthermore, the restricted cubic splines (RCS) were applied to explore potential nonlinear relationships between them.

**Result:**

The average age of participants was 68 years (interquartile range [IQR]: 63–74), and 537 (48.6%) participants were male. Logistic regression analysis showed no significant association between ApoB and CI (OR: 1.003, 95% CI: 0.998–1.008, *p* =  0.215), while a significant association was observed between LDL‐C/ApoB and CI (OR: 1.519, 95% CI: 1.206–1.922, *p* < 0.001). The results remained consistent after adjusting for gender and age (OR: 1.349, 95% CI: 1.062–1.718, *p* < 0.05). Further RCS analysis indicated a nonlinear relationship between LDL‐C/ApoB and CI (P_non‐liner_ < 0.0001), even after adjusting age and gender (P_non‐liner_ < 0.0001).

**Conclusion:**

Our study demonstrates that the LDL‐C/ApoB ratio is a potential risk factor for CI in the elder population. Thus, maintaining an optimal LDL‐C/ApoB ratio might be crucial in preventing CI.

